# Liquid Biopsy for the Diagnosis of Viral Hepatitis, Fatty Liver Steatosis, and Alcoholic Liver Diseases

**DOI:** 10.3390/ijms21103732

**Published:** 2020-05-25

**Authors:** Ciniso Sylvester Shabangu, Jee-Fu Huang, Hui-Hua Hsiao, Ming-Lung Yu, Wan-Long Chuang, Shu-Chi Wang

**Affiliations:** 1 Department of Medical Laboratory Science and Biotechnology, Kaohsiung Medical University, Kaohsiung 80708, Taiwan; u107567006@kmu.edu.tw; 2 Center for Cancer Research, Kaohsiung Medical University, Kaohsiung 80708, Taiwan; jf71218@gmail.com (J.-F.H.); fish6069@gmail.com (M.-L.Y.); 3 Center for Liquid Biopsy, Kaohsiung Medical University, Kaohsiung 80708, Taiwan; huhuhs@cc.kmu.edu.tw; 4 Hepatobiliary Division, Department of Internal Medicine, Kaohsiung Medical University Hospital, Kaohsiung Medical University, Kaohsiung 80708, Taiwan; waloch@kmu.edu.tw; 5 Faculty of Internal Medicine, School of Medicine, College of Medicine, Kaohsiung Medical University, Kaohsiung 80708, Taiwan; 6 Hepatitis Research Center, Kaohsiung Medical University, Kaohsiung 80708, Taiwan

**Keywords:** viral hepatitis, non-alcoholic fatty liver disease, alcoholic liver disease, biomarkers, extracellular vesicles, microparticles, exosomes

## Abstract

During the progression from hepatitis to fibrosis, cirrhosis, and liver failure, the accumulation of stressed/damaged hepatocyte elements associated with liver inflammation is critical. The causes of hepatocyte injuries include viral hepatitis infections, alcoholic hepatitis, and non-alcoholic fatty liver disease. Hepatocyte-derived extracellular vesicles (Hep-EVs) released from stressed/damaged hepatocytes are partly responsible for liver disease progression and liver damage because they activate non-parenchymal cells and infiltrate inflammatory cells within the liver, which are in turn are an important source of EVs. This cell-to-cell signaling is prevalent during inflammation in many liver diseases. Accordingly, special emphasis should be placed on liquid biopsy methods for the long-term monitoring of chronic liver diseases. In the present review, we have highlighted various aspects of current liquid biopsy research into chronic liver diseases. We have also reviewed recent progress on liquid biopsies that focus on cell-free DNA (cfDNA), long non-coding RNA (lncRNA), and the proteins in EVs as potential diagnostic tools and novel therapeutic targets in patients with viral hepatitis, fatty liver steatosis, and alcoholic liver diseases.

## 1. Introduction

Chronic liver disease is a major public health issue globally. It includes chronic viral hepatitis, fatty liver disease, among other causes, and accounts for approximately 3.5% of deaths [1]. Viral hepatitis is a persistent state of liver inflammation caused by various viruses. It increases the risk of cirrhosis and hepatocellular carcinoma (HCC). Chronic hepatitis B virus (CHB) and chronic hepatitis C virus (CHC) infections are major risk factors for HCC, and the association has ranged from 45 to 80% in various regional epidemics. In recent decades, non-alcoholic fatty liver disease (NAFLD) and alcohol-associated liver disease have emerged as urgent etiologies associated with an increasingly westernized global lifestyle. It is worth noting that the increase in non-alcoholic steatohepatitis (NASH) associated with obesity and metabolic syndrome may have led, directly or indirectly, to an increase in the incidence of HCC [2]. NAFLD-associated HCC presents as mild stage fibrosis but it is linked to advanced HCC stage and poor overall survival [3,4]. Because most NAFLD patients are asymptomatic, a feasible follow-up schedule and an HCC surveillance strategy remain elusive owing to the potential risk of cirrhosis, HCC, and liver failure in certain patients [5].

Liver biopsy remains the gold standard for the diagnosis of various chronic liver diseases, and for determining the patterns and severity of liver injury, inflammation, and fibrosis stage. However, liver biopsy is invasive, and is prone to sampling errors during the investigation of fat deposition in hepatocytes. It also carries the potential risk of bleeding. Moreover, despite intensive research, currently available non-invasive blood tests are not sufficiently sensitive or specific and are therefore of limited use. Various imaging modalities—including magnetic resonance imaging (MRI)-based and ultrasound-based elastography—are used for the assessment of liver steatosis and fibrosis, but there is much room for improvement with regard to diagnostic accuracy, especially in the early stages of fibrosis [6]. Biomarkers that can redress these shortcomings might provide significant advances in the diagnosis and monitoring of disease progression and regression in clinical settings. Liquid biopsy has potential as a less invasive alternative to tissue biopsy. It addresses several unmet clinical needs, including sensitivity, specificity, the determination of prognoses, and the prediction of therapeutic responses.

Liquid biological specimens comprising elements such as cell-free DNA (cfDNA) from intact blood, extracellular vesicles (EVs), and long non-coding RNA (lncRNA) are potential tools for monitoring tumor dynamics in terms of the early detection of de novo growth and recurrence [7]. EVs are microscopic capsules bound by membranes that comprising variable of contains by organs [8]. The three main types of EVs are microparticles/microvesicles (MPs/MVs), exosomes, and apoptotic bodies, which are released from disintegrating or activated cells. They are generated in different ways and vary in size and composition. In general, MPs/MVs are 100–1000 nm in diameter and are directly generated from the plasma membrane by a budding process [9]. Apoptotic bodies are up to 500 nm in diameter and contain cellular fragments [10]. Exocytosis is a cellular process that involves the release of multiple vesicles (40–150 nm in diameter) known as exosomes, which are formed by the fusion of multivesicular bodies with the plasma membrane [11]. EVs contain various cellular components including proteins, DNA molecules (double-stranded DNA (dsDNA), single-stranded DNA (ssDNA), and mitochondrial DNA (mtDNA)), RNA molecules (messenger RNA (mRNA), microRNA (miRNA), and lncRNA), and lipids [12]. 

Some of the protein cargoes delivered by EVs from hepatocytes are implicated in various pathways, including those involving detoxification, endosomes, cytosolic proteins, apolipoproteins, and coagulation-associated proteins. MPs/MVs have been found to contain typical markers such as actinin-4, mitofilin, and phosphatidylserine. Exosomes contain a rich variety of components, such as heat shock proteins (HSP60, HSP70, HSP90), tetraspanins (CD9, CD10, CD26, CD63, CD81, CD82), and integrins [8]. Sphingosine kinase 2 (SK2) is delivered by exosomes that are formed by hepatocytes and target other hepatocytes; it catalyzes the formation of sphingosine-1-phosphate (S1P), implying that it is a disease-specific cargo of hepatocyte-derived exosomes [13]. EVs are formed by various types of liver cells, such as hepatocytes, hepatic stellate cells (HSCs), endothelial cells, Kupffer cells, and natural killer cells. They act as essential intracellular communication regulators, and are associated with various vital biological processes, including inflammation and tissue remodeling. EVs are expelled into the extracellular space by various types of liver cells and are thereby introduced into the circulation. Long non-coding RNAs (lncRNAs)—which regulate biological and genetic functions—have been identified in EVs. lncRNAs have been implicated in numerous biological disorders because they regulate developmental processes. As wells as secreting the components described above into the circulating blood, cells can also export the precursors produced by pathogens such as viruses during their replication. One example of this phenomenon is provided by the detection of covalently closed circular DNA (cccDNA) and pre-genomic RNA (pgRNA) formed during the replication of hepatitis B virus (HBV). The assembly of proteins and nucleic acids originating from EVs also can change in response to various stimulations. Therefore, EVs derived from cells or organs under stress or affected by disease are potential biomarkers as they are different from those secreted during healthy homeostasis [12,14]. Liquid biopsy methods are now used widely to detect many aspects of chronic liver diseases. They are informative and provide in-depth information about the diverse spectrum of disease progression.

## 2. Liquid Biopsies for Viral Hepatitis

### 2.1. Liquid Biopsies Associated Viral Hepatitis Infections

Viral hepatitis is an inflammation of the liver associated with infection by a hepatitis virus. Researchers have debated whether viruses have infection strategies that integrate EV-regulated communication pathways [15]. For example, enveloped viruses use the phosphatidylserine receptor to identify and enter target cells [16,17]. Phosphatidylserine groups have been identified on the surfaces of exosomes in numerous studies. This suggests that hepatitis viruses may incorporate themselves into EVs during biogenesis to enhance viral spread, as indicted by cell models and serum samples [18,19,20].

**Figure 1:**
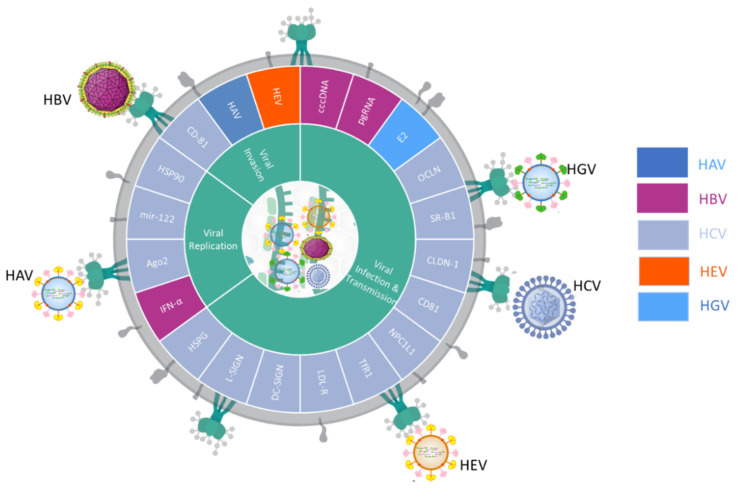
Summary of the viral liquid biopsy markers driven by hepatitis viruses in the pathogenesis of viral hepatitis. Classification of viral related liquid biopsies by viral infection and transmission, viral replication, and viral invasion on the HAV (◼), HBV(◼), HCV(◼), HEV(▮), and HGV(▮).

Hepatitis A—which is a picornavirus—uses the host’s EV machinery to propagate enveloped hepatitis A (eHAV); it comprises of two components: a nonenveloped form and a virus-like particle structure derived from the host membrane that is similar to an exosome. eHAV virions are further implicated in the ESCRT (endosomal sorting complexes required for transport) pathway, which promotes the biogenesis of multivesicular bodies (MVBs) [21]. Study found that eHAV virions resemble enveloped viruses and are infectious; they may be transported via an exosome-like system in which the HAV capsids are packaged as cargo into MVBs, and eventually enter the blood [22]. This indicates that encapsulated viral vesicles, like exosomes, are protected from antibody-mediated neutralization [21]. HBV depends on the endosomal mechanisms of the host cell to produce intraluminal vesicles (ILVs) and MVBs [23]. EV fragments are transported and delivered by MVBs. Simultaneously, machinery perturbation arrests viral maturation by blocking the encapsulation of viral envelope proteins derived from solvent-insoluble membranes. This may hinder viral propagation. As with EVs, hepatitis C virus (HCV) targets are determined by multiple factors. The HCV viral entry mechanism has evolved considerably and requires numerous molecules including Heparan sulfate proteoglycans (HSPG), liver/lymph node-specific intracellular adhesion molecules-3 grabbing non-integrin (L-SIGN), Dendritic cell–specific ICAM-3 grabbing-nonintegrin (DC-SIGN), low-density lipoprotein receptor (LDL-R), Transferrin Receptor (TfR1), NPC1 Like Intracellular Cholesterol Transporter 1 (NPC1L1), Cluster of Differentiation 81 (CD81), Claudin 1 (CLDN-1), Scavenger receptor class B type 1 (SR-B1), and Occludin (OCLN), which illustrates the intricacy of the EV entry mechanism [24]. It has recently been shown that CD81-rich exosomes derived from HCV-exposed cells can trans-infect naive human hepatoma cells among neutralizing antibodies [25]. In vivo studies have also confirmed that anti-CD81 antibodies can suppress viral infection [26,27]. Moreover, EVs isolated from the sera of CHC patients not only contain HCV RNA and independently regulate HCV replication via viral receptors, but are also corelated with HSP90, Argonaute RISC Catalytic Component 2 (Ago2), and mir-122, which increase HCV replication by EV-regulated HCV transmission to naive cells [25]. GB virus C (GBV-C), formally known as hepatitis G virus (HGV), is a member of the *Flaviviridae* family. It has been shown that GB virus C particles inhibit T cell activation via envelope E2 protein-mediated inhibition of T-cell receptor (TCR) signaling [28]. These findings suggest that EVs play various functions in the pathogenesis of viral hepatitis as shown in Figure 1.

### 2.2. Liquid Biopsies Associated Viral Hepatitis Immunity

EVs also allow HCV to evade immunity by protecting the HCV RNA from degradation. The hepatitis A virus evades the human immune response by becoming encapsulated in the membranes of the host immune cells [29]. HCV-stimulated monocytes differentiate into polarized M2 macrophages; this activates HSCs and stimulates the secretion of EVs from HCV-infected hepatocytes [30]. Liver injury may stimulate the differentiation of monocytes into macrophages and hepatic macrophage recruitment. Elevated numbers of platelet-derived EVs are also associated with liver fibrosis biomarkers, such as serum hyaluronate and the N-terminal propeptide of type III procollagen [31]. Patients with chronic HCV infection experience greater platelet activation and increased levels of circulating platelet-derived EVs compared to those with chronic HBV infection. Immune cell-derived circulating EVs are associated with advanced liver disease and may be identified by microparticle markers of cell type: T cells by CD4+/8+; monocytes by CD14+; neutrophils by CD15+; platelets by CD41+; and invariant natural killer T (iNKT) cells by Valpha24/Vbeta11 [32]. CHC patients can be identified among chronic hepatitis patients by cell-derived EV biomarkers. The authors of another study found that liver cells exposed to IFN-α conferred resistance to HBV replication via cell-to-cell communication through EVs in infected liver cells [33]. The immune cells are implicated in liver inflammation and liver-related diseases by host immune system regulation and alteration of the microenvironment [34].

### 2.3. Biomarkers of Liquid Biopsies Associated Viral Hepatitis

Nucleic acids and viral hepatitis-associated proteins have been identified in EVs from the sera of patients with chronic viral infections [35]. Long non-coding RNAs (lncRNAs) can affect the regulation of gene expression and have an impact on many different cellular processes. Xu et al. demonstrated significantly higher levels of exosomal HNRNPH1 in HBV-associated HCC patients with vascular invasion and lymph node metastasis than those in non-HCC patients [36]. A positive correlation among lncRNA-HULC, lncRNA-HEIH, and hepatitis B X-interacting protein (HBXIP) indicated that hepatitis B X protein (HBx) may alter lncRNA expression, which in turn may promote HCC development [37]. lncRNA-HULC modulates microRNA-539 (miR-539), which downregulates APOBEC3B, thereby promoting HBV replication [38]. Both serum and exosomal lncRNA-HEIH expression levels increase in HCV-related HCC patients, whereas serum lncRNA-HEIH expression levels are significantly lower than exosomal lncRNA-HEIH expression levels in CHC patients [39]. These data suggest that lncRNA is a potential liquid biotarget for HBV-related HCC. MHC class I chain-related A (MICA) is another HCV-associated liquid biopsy target. High levels of soluble MICA (sMICA) have been identified in the sera of HCV-induced HCC patients bearing the G allele as opposed to the A allele as a result of the single nucleotide polymorphism (SNP) rs2596538 [40]. There was an increase in the risk of liver fibrosis in the CHC patients with the G allele, whereas the levels of sMICA were elevated in the HCV-associated HCC patients following viral eradication [41,42]. A genome-wide association study (GWAS) demonstrated that elevated serum levels of MICA and soluble MICA (sMICA)—encoded by the G allele resulting from SNP rs2596542—increased the risk of HBV-associated HCC [43].

Viral amplification and replication could be used in target detection. Chronic HBV infection is characterized by a persistent episomal viral genome, namely covalently closed circular DNA (cccDNA). cccDNA constitutes a stable mini-chromosome within the infected hepatocyte nucleus [44]. The persistence of cccDNA and the inability of the immune system to eradicate the virus contribute to the failure of viral clearance and to relapse, even after treatment goals have been achieved. Therefore, true HBV clearance or functional cure is generally defined as the complete elimination of cccDNA from infected hepatocytes [44]. Theoretically, even if one molecule of HBV cccDNA with replication potential remains in a liver cell, there is still a risk of virological recurrence after ceasing antiviral therapy. The presence of pre-genomic RNA (pgRNA) in mature HBV viral particle nucleocapsids is linked to the persistence of viral infection and the risk of virological recurrence. Lin et al. demonstrated the use of real-time quantitative polymerase chain reaction (qPCR) to determine HBV pgRNA levels in the sera of HBV-infected patients, and to make a better assessment of changes in the sustained viral response (SVR) [45]. Regarding pathogenic cell-free genomics, Tokuhisa et al. were the first to demonstrate that the levels of circulating cfDNA increase in the sera of patients with HCV-related HCC [46]. Iida et al. also reported an association between the level of cfDNA in the serum and the metastatic capability of HCV-related HCC, which suggests the potential of cfDNA as an active metastasis biomarker following curative surgery [47]. Patients with CHC-related HCC have 3–4 times higher levels of cfDNA in their sera or plasma than their non-HCC CHC counterparts, and the level is 20 times higher than in healthy controls [48]. The viral hepatitis-related liquid biomarkers are listed in Table 1.

## 3. Liquid Biopsies for the Fatty Liver Disease

### 3.1. Biomarkers Associated Non-Alcoholic Steatohepatitis/Non-Alcoholic Fatty Liver Disease (NASH/NAFLD)

NAFLD is a well-known liver disorder, with 25% prevalence across the globe. It is broadly distributed in diverse populations and regions. NASH is the severe form of NAFLD, which is characterized by lobular inflammation or the chronic low-grade proinflammatory state of hepatocytes with or without fibrosis. NAFLD is currently the most prevalent cause of liver transplantation [49]. The prevalence of NAFLD has increased rapidly over the past few decades in the Asia–Pacific region, at a comparable rate to westernization. NAFLD is an intermediate stage in various progressive diseases that are not associated with high alcohol intake. Clinical manifestations include confined intrahepatic triglyceride accumulation (exceeding 5%) and necroinflammation of the hepatocytes, with some patients progressing to fibrosis, cirrhosis, and HCC. Lipotoxicity develops during the progression of NAFLD owing to the accumulation of toxic lipids and stressed/damaged hepatocytes; it is linked to metabolic disorders including dyslipidemia, type 2 diabetes, and obesity [50].

Various mechanisms contribute to the intrahepatic accumulation of fat. These include fluctuations in the level of fatty acids, increased lipogenesis, the secretion of very-low-density lipoprotein (VLDL), and suppressed clearance through β-oxidation [51]. NASH is characterized by an increase in the level of serum triacylglycerols (TAGs), and a positive hepatic histological index distinguishes NASH in NAFLD patients [52]. Minimal TAG levels may be hepato-protective, whereas prolonged lipid storage may escalate into inflammation and metabolic dysfunction [53]. The accumulation of TAGs—which occur predominantly as macrovesicular lipid droplets in hepatocytes—is associated with liver injury or necroinflammation [54]. One study using plasma samples demonstrated that peroxisome proliferator-activated receptor gamma (PPARγ) DNA methylation is associated with the fibrosis index and may be used as a severity predictor in NAFLD diagnoses [55]. The authors of another study found that homocysteine (Hcy) serum concentrations are significantly higher in NAFLD patients and are not influenced by chronic hepatitis [56]. Furthermore, there is a positive correlation between plasma Hcy levels and the severity of steatosis; therefore, plasma Hcy levels could be used to discriminate between NASH and simple steatosis [57]. Glutamine, glycine and serine which are precursors of antioxidants such as glutathione (GSH) were demonstrated to be decreases in the NAFLD patients compared to healthy individuals [58]. Proline and hydroxyproline, the elements of collagen protein, also observed to be elevated in serum of patients with NAFLD compared to healthy individuals [59]. Another study indicated that a rate-limiting enzyme Acetyl-CoA carboxylase (ACC) increased expression in NAFLD animal models [60]. Indeed, Alkaline phosphatase (ALKP) elevated levels are associated with hepatic fibrosis in steatohepatitis patients. ALKP serum level are notably higher in NASH patients compared to those without NASH [61].

### 3.2. Hepatocyte-Derived Vesicles Associated Non-Alcoholic Steatohepatitis/Non-Alcoholic Fatty Liver Disease (NASH/NAFLD)

With regards to NAFLD, EVs play a key role in the mechanism of liver damage and disease progression through the accumulation of lipotoxic lipids in hepatocytes. In turn, hepatocyte-derived extracellular vesicles (Hep-EVs)—which are discharged from damaged or stressed hepatic cells—exacerbate the progression of liver disease through the stimulation of non-parenchymal cells [62]. In mouse hepatocytes and human hepatocyte-derived carcinoma cell (HuH) models, palmitate and lysophosphatidylcholine (LPC) increase the release of EVs, demonstrating the conservation of toxic-lipids and cytochrome P450 2E1 (CYP2E1) within serum EVs. LPC-induced hepatic lipotoxicity is triggered by chemokine (C-X-C motif) ligand 10 (CXCL10), which is enriched in hepatocyte-derived vesicles, and promotes macrophage chemostasis [63]. This suggests that hepatocyte-derived EVs are potential biomarkers of NASH [64]. In addition, lipotoxic EVs from hepatocytes stimulate pro-fibrogenesis in hepatic stellate cells [59]. With regard to NASH, lipids facilitate the release of EVs through the tumor necrosis factor receptor superfamily member 10B (TNFRSF10B) signaling pathway in human and mouse hepatocytes [65]. The study demonstrated that the upregulation of toll-like receptor 9 (TLR9) pathway was activated by the high levels of hepatocyte mtDNA in the MPs of mouse plasma to induces lipotoxicity and inflammation in NASH patients [66].

### 3.3. Liquid Biopsies Associated Chemoattraction in Non-Alcoholic Steatohepatitis/Non-Alcoholic Fatty Liver Disease (NASH/NAFLD)

**Figure 2:**
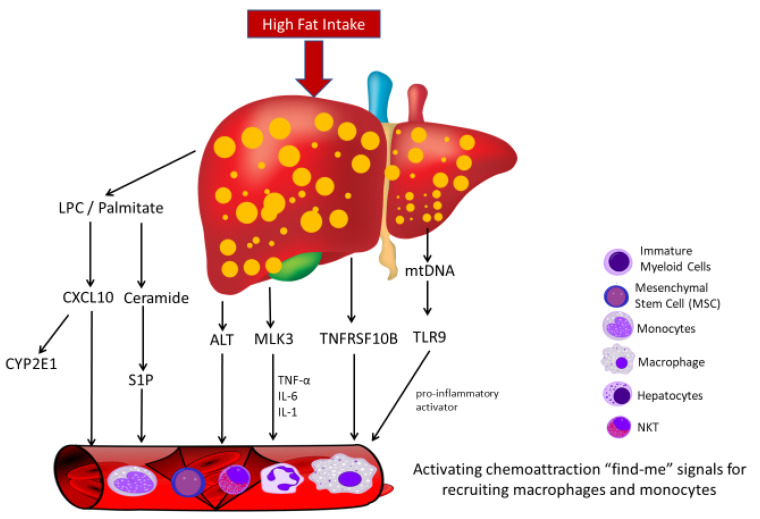
Summary of the liquid biopsies activating chemoattraction signal pathways for recruiting immune cells in NASH/NAFLD. palmitate and lysophosphatidylcholine (LPC)-induced hepatic lipotoxicity is triggered by CXCL10 with hepatocyte-derived CYP2E1 vesicles enrichment; Palmitate-stimulated EVs are enriched in ceramide (C16:0)-derived sphingosine 1 phosphate (S1P) to chemoattract macrophages. Increased numbers of EVs derived from invariant natural killer T cells and CD14+ monocytes are associated with alanine transaminase (ALT) level in serum. Mlk3−/− mice fed a diet that was high in fats and carbohydrates exhibited suppressed cytokine expression and macrophage infiltration compared to wild-type (WT) mice. Lipid-induced TNFRSF10B signaling causes release of inflammatory extracellular vesicles from hepatocytes. High levels of hepatocyte mtDNA in the microparticles (MPs) activated toll-like receptor 9 (TLR9) pathway for chemotaxis of neutrophils and M1 macrophages.

EVs play a role in cell-to-cell communication by transferring biologically active molecules into target cells while regulating NASH pathogenesis and progression. The fatty liver disease-related liquid biomarkers are listed in Table 2. Macrophage activation and influx in the liver are important for the progression of NAFLD because hepatic macrophages promote NASH development via cytokines IL-1, IL-6, and TNFα [67]. A model comprising Mlk3−/− mice fed a diet that was high in fats and carbohydrates exhibited suppressed cytokine expression and macrophage infiltration compared to wild-type (WT) mice [68]. This indicates that non-parenchymal cells and infiltrated inflammatory cells are optional yet important sources of EVs that perpetuate liver injury. The TLR9 is facilitated by the chemotaxis of neutrophils and M1 macrophages—proves that TLR9 is a pro-inflammatory activator in NASH [69]. The production of Hep-EVs increases in hepatocytes treated with palmitic acid (PA), and hepatic stellate cells (HSCs) are stimulated when treated with exosomes from PA-treated hepatocytes in a cell culture model [70]. Exosomes released by healthy or compromised hepatocytes and Hep-EVs are crucial in cell-to-cell communication, especially in hepatocytes and hepatocyte-to-HSC signaling. EVs derived from hepatocytes containing excessive levels of lipids promote liver fibrosis through pro-inflammation, which directly affects immune cells. Kornek et al. reported increased numbers of EVs derived from invariant natural killer T cells and CD14+ monocytes—which are associated with alanine transaminase (ALT)—in plasma samples from NAFLD patients [32]. There is an increase in inflammatory cell-derived Hep-EVs in NAFLD patients and in patients with either or both of alcohol-related cirrhosis and chronic hepatitis C-related cirrhosis [32,71]. Immature myeloid cells (CD11b^+^, Ly6C^hi^, Ly6G^-^) have also been detected in a high-fat diet mouse model of NASH [72]. Furthermore, mesenchymal stem cell (MSC)-derived EVs are linked to liver inflammation and NASH progress, and are associated with increased proinflammatory markers (TNF-α, IL-1β, IL-6, and IL-12) [73]. Mouse obesity models have been used to demonstrate that levels of circulating adipocyte-derived microparticles are high in stressed adipocytes, which secrete chemoattraction “find-me” signals to recruit macrophages and monocytes [74]. Notably, palmitate-stimulated EVs are enriched in ceramide (C16:0). EVs chemoattract macrophages via ceramide-derived sphingosine 1 phosphate (S1P), which leads to macrophage activation [75]. This result also indicated that the amino acid metabolomics also can be detected by the liquid biopsies and significantly associated with the progression of liver fibrosis [76]. To date, the literature indicates that the EVs secreted by various cells play a crucial role in inflammatory regulation in advanced steatosis. However, the major components of EVs that are involved in cell or cytokine activation require further investigation. Figure 2 is an illustration of the NAFLD/NASH effects on the liver driven by lipid accumulation and their effects.

## 4. Liquid Biopsies for Alcoholic Liver Disease (ALD)

### 4.1. Biomarkers Associated Alcoholic Liver Disease (ALD)

**Figure 3:**
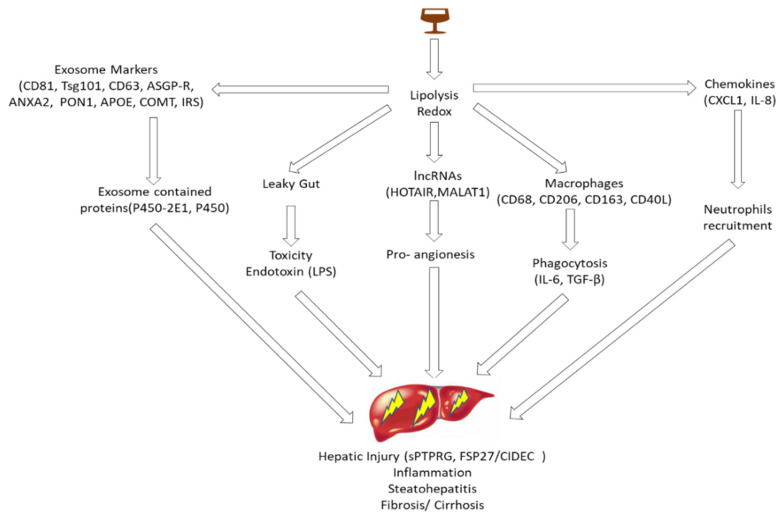
The summary of the liquid factors associated with liver inflammation in Alcoholic liver disease (ALD).

Alcoholic liver disease (ALD) foreshadows a wide variety of liver pathologies from basic steatosis to advanced liver injuries such as steatohepatitis and fibrosis/cirrhosis [77]. Steatosis almost always occurs in the hepatocytes of people that consume excessive amounts of alcohol. In 20–40% of heavy drinkers there is progression to steatohepatitis through neutrophil infiltration and related inflammation [78]. Excessive alcohol consumption promotes the production of prooxidants and strengthens the antioxidant mechanism, which leads to hepatocyte injury accompanied by inflammation; this eventually results in liver failure or cancer [79]. ALD hepatitis is directly related to the toxic effects of alcohol. It is indirectly caused by fecal infection or adipose tissue impairment resulting from bacteria-related endotoxins, including lipopolysaccharides (LPSs). It is also caused by elevated levels of free fatty acids, and by proinflammatory adipokines, which are found in the blood but also regulate liver function. Several authors have reported changes in the levels of ALD-corelated proteins in the serum. TG accumulation in the liver is the first histological change due to alcohol, and is reversible. Trinchet et al. (1994) reported that elevated levels of primary bile acids are associated with alcoholic hepatitis (AH) [80]. Moratti et al. reported that increased plasma levels of protein tyrosine phosphatase receptor type gamma (PTPRG) isoform sPTPRG in EVs excreted from the human hepatocellular carcinoma cell line HepG2 are associated with alcohol-related hepatic injury [81]. FSP27/CIDEC (fat-specific protein 27/cell death-inducing DNA fragmentation factor alpha-like effector c)—which promotes alcohol-stimulated liver damage—was highly expressed in the hepatocytes of a mice model following chronic and binge alcohol consumption, and in alcoholic hepatitis (AH) patients [82]. The summary of the liquid factors associated with liver inflammation in ALD as shown in Figure 3 in advance.

### 4.2. Hepatocyte-Derived Vesicles Associated Alcoholic Liver Disease (ALD)

Investigations into EVs as biomarkers for alcoholic hepatitis in humans have revealed increased levels of EVs in alcoholic hepatitis patients and patients who consume excessive amounts of alcohol [83]. A study involving alcohol-fed rodents and human patients who consumed alcohol revealed elevated levels of cytochrome P450-2E1 and cytochrome P450 isoforms in exosomes. The authors further reported an increase in the number of EVs involving CYP2E1 and P450 isoforms that was associated with an increase in oxidative stress in the endoplasmic reticulum [84]. Such endotoxins in the circulation increase the likelihood of progression to advanced-stage disease through fecal infection [85]. EV levels are elevated in patients with a history of alcohol abusive compared to those that consume extreme levels of alcohol and those that do not drink. EV levels are positively associated with ALT serum levels, mtDNA serum levels, and circulating neutrophil levels [86]. The injection of heat shock protein 90-containing EVs—which were isolated from the sera of alcohol-consuming mice—into naive mice demonstrated the internalization of injected EVs in hepatocytes; this resulted in elevated CCL2 expression [87], which indicates that alcohol activates an innate immune response through hepatocyte-derived EVs.

Hepatocytes secrete a variety of EV proteins including exosomal marker proteins (tumor Susceptibility 101 (Tsg101), Cd63, and Cd81); hepatic-specific proteins such as asialoglycoprotein receptor (ASGP-R); and disease-related proteins such as annexin A2 (ANXA2), paraoxonase-1 (PON1), apolipoprotein-E (APOE), catechol O-methyltransferase (COMT), and insulin receptor substrate (IRS). Exosome membrane proteins contain functional features that stimulate the expression of non-exosome proteins in exosomes [12]. In a proteomic analysis of DGAL- or LPS-exposed rats, the serum EVs had a distinct protein composition associated with liver toxicity, which suggested their potential use as liver injury biomarkers [88]. EV albumin protein concentrations are correlated with APAP dosage and exposure during serum ALT alterations. Therefore, EV proteins may be ideal biomarkers for drug-induced liver injury [89]. Using patient cohorts, Bissonnette et al. demonstrated increased cytokeratin-18 (CK-18) fragments in hepatocyte-derived cytokeratin-18 (CK-18) fragments M30 and M65 in EVs [90]. The severity of alcoholic hepatitis is linked to EVs containing CD34+ and ASGPR+ [91].

### 4.3. Liquid Biopsies of Markers Associated with Inflammation in Alcoholic Liver Disease (ALD)

The accumulation of hepatic macrophages has been observed in the portal tracts of ALD patients. EVs stimulate the activation of macrophage activation and the induction of inflammatory cytokines, as demonstrated in an unbiased microarray-based antibody neutralization experiment [92]. Alcohol-exposed, monocyte-derived EVs activate naive monocytes to polarize M2 macrophages by increasing the expression of surface receptors CD68, CD206, and CD163, and the secretion of IL-10 and TGFβ, which elevates phagocytic activity [89]. Furthermore, the levels of CD40L-rich EVs were elevated in the sera of AH patients. The proteins detected within EVs during macrophage activation suggests that CD40L may stimulate the activation of macrophages in experimental alcoholic hepatitis models [92]. Chemotaxis is the initiating step in neutrophil recruitment, and requires chemokines such as IL-8, which is essential for neutrophil recruitment [93]. IL-8 levels are extremely elevated in both circulating and hepatic neutrophils, which are associated with alcoholic hepatitis and disease aggression [94,95,96]. CXCL1 is an IL-8 homologue that has been demonstrated to promote the recruitment of neutrophils in a mice model. Studies in a mouse model of ALD have shown that elevated levels of CXCL1 in the serum and hepatocytes exacerbate liver injury [58,97]. In endothelial cell-derived extracellular vesicles, ethanol stimulates the upregulation of lncRNAs HOTAIR and MALAT1, which facilitates the pro-angiogenic effects of endothelial-derived vesicles [98]. The alcoholic hepatitis-related liquid biomarkers are listed in Table 3.

## 5. Conclusions

**Figure 4:**
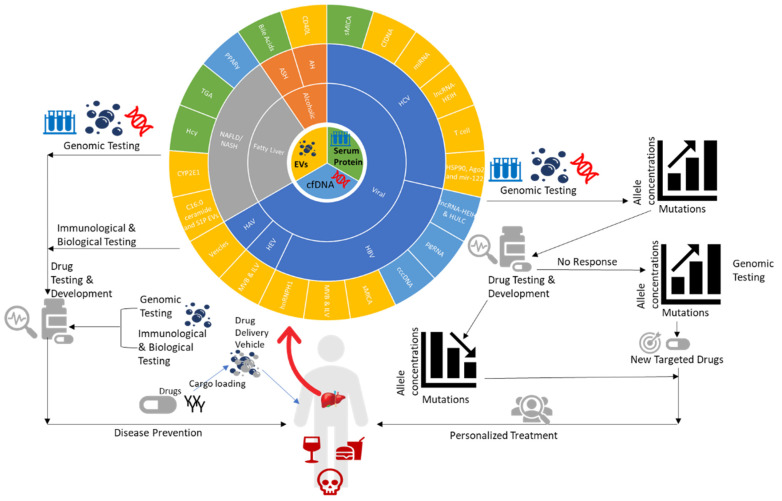
The potential of the liquid biopsy as a non-invasive and accurate approach to the diagnosis and monitoring of chronic liver disease by disease associated markers from EVs, cfDNA, and serum protein. Biomarks have potential for further gene allele and mutation study to design the genomic testing depends on the gene expression and sequencing result for personalized therapeutic strategy. The biomarks expression testing level not only to monitor the disease process, also can apply for the drug design, drug testing and therapeutic efficiency in the future.

In the present review, we have highlighted the use of virus-related cfDNA, proteins and lncRNA in EVs/EPs and exosomes for liquid biopsies for the diagnosis of chronic liver diseases (Figure 4). Numerous studies have demonstrated that EVs affect the chronic inflammatory response in the progression of liver diseases. EVs released from stressed hepatocytes are fundamental modulators of these responses and take effect through various actions in different target cells including HSCs, MSCs, and macrophages; they are implicated in complex networks of cell-to-cell communication in liver disease development and progression. Some studies have presented analyses of EVs as carriers of specific proteins or genes involved in the biogenesis of diseases which can provide therapeutic targets in the future. This indicates the potential of the liquid biopsy as a non-invasive and accurate approach to the diagnosis and monitoring of chronic liver disease.

## Figures and Tables

**Table 1:** Liquid biomarkers of viral hepatitis liver diseases.

References	Biomarker	Type of Liquid Biopsy	Application	Disease	Source	Sample Type
McKnight, Xie et al. [22]	MVB	Exosomes	Viral infection	HAV	Infected cells	Serum
J Gen Virol 2014, Nagashima S. et al. [23]	MVB	Exosomes	Viral infection	HEV	Infected cells	Cell supernatants
PLoS Pathog 2014, Bukong TN. et al. [25]	HSP90, Ago2, and mir-122	Exosomes	Viral infection	HCV	Infected cells	Serum/Cell supernatants
Clin Chem Lab Med. 2018, Xu H. et al. [36]	Exosome-hnRNPH1	Exosomes	Biomarker	HBV	Hepatocyte	Serum
Gastroenterol. Res. Pract. 2018, Ruan, L. et al. [37]	lncRNA-HEIH & lncRNA-HULC	-	Biomarker	HBV	Hepatocyte	Peripheral blood
Cancer Biomark 2018, Zhang C. et al. [39]	lncRNA-HEIH	Exosomal	Biomarker	HCV	Hepatocyte	Serum
J Gastroenterol Hepatol 2019, Huang CF. et al. [41]	sMICA	Protein	Biomarker	HCV	Infected cells	Serum
PLoS One. 2012, Kumar V. et al. [43]	sMICA	protein	Biomarker	HBV	Hepatocyte	Serum
Viruses. 2017, Allweiss L. et al. [44]	cccDNA	Viral DNA	Diagnosis	HBV	Virus	Serum/Cell supernatants
Journal of Clinical Microbiology 2019, Lin N. et al. [45]	pgRNA	Viral RNA	Diagnosis	HBV	Virus	Serum
Br J Cancer 2007, Tokuhisa Y. et al. [46]	cfDNA	HCC DNA	Biomarker	HCV	Hepatocyte	Serum/plasma
Gastroenterology 2012, Kornek M. et al. [32]	Levels	Microparticles	Hepatic inflammation	HCV	-	Serum/plasma

Multivesicular body (MVB); Cell-free DNA (cfDNA); covalently closed circular DNA (cccDNA); extracellular vesicle (EV); hepatitis A virus (HAV); hepatitis B virus (HBV); hepatitis C virus (HCV); hepatitis G virus (HGV); human hepatocyte-derived carcinoma cells (HuH); intraluminal vesicle (ILV); long non-coding RNA (lncRNA); MHC class I chain-related A (MICA); multivesicular body (MVB); peripheral blood mononuclear cell (PBMC); pre-genomic RNA (pgRNA); single nucleotide polymorphism (SNP); soluble MICA (sMICA).

**Table 2:** Liquid biomarkers of NASH diseases.

References [ref]	Biomarker	Type of Liquid Biopsy	Application	Disease	Source	Sample Type
Hepatology. 2016, Ibrahim, S.H. et al. [63]	Toxic-Lipids and CYP2E1	EVs	Prognostic Biomarker	NASH	Hepatocyte	Cell Supernatants/Mouse Model Serum
Clin Sci (Lond) 2017, Mridha, Haczeyni et al. [69]	TLR9	EVs	Diagnostic & prognostic biomarker	NASH	Hepatocytes	Cell supernatants
J Lipid Res 2016, Kakazu E. et al. [75]	C16:0 ceramide and S1P	EVs	Diagnosis & monitoring biomarker	NASH	Hepatocytes	Mouse model Serum
Gastroenterology 2012, Kornek M. et al. [32]	Levels	Microparticles	Hepatic inflammation	NAFLD	-	Serum/plasma
J Clin Invest 2016, Garcia-Martinez, Santoro et al. [66]	mtDNA	Microparticles	Diagnostic & prognostic biomarkers	NASH	Hepatocytes	Plasma
Sci Rep. 2017, Yang RX. et al. [52]	Triacylglycerol	Protein	Diagnosis biomarker	NAFLD	-	Serum
J Gastroenterol Hepatol. 2005, Gulsen, M. et al. [57]	Hcy	Protein	Predictor biomarkers	NAFLD	Hepatocytes	plasma
Gut. 2017, Hardy T. et al. [55]	PPARγ	DNA methylation	Prognostic biomarker	NAFLD	-	Plasma

Extracellular vesicles (EVs); cytochrome P450 2E1 (CYP2E1); mitochondrial DNA (mtDNA); non-alcoholic fatty liver disease (NAFLD); non-alcoholic steatohepatitis (NASH); peroxisome proliferator-activated receptor gamma (PPARγ); toll-like receptor 9 (TLR9); sphingosine 1 phosphate (S1P). homocysteine (Hcy); cytochrome P450 2E1 (CYP2E1); mitochondrial DNA (mtDNA); non-alcoholic fatty liver disease (NAFLD); non-alcoholic steatohepatitis (NASH); peroxisome proliferator-activated receptor gamma (PPARγ); toll-like receptor 9 (TLR9).

**Table 3:** Liquid biomarkers of alcoholic liver diseases.

References [ref]	Biomarker	Type of Liquid Biopsy	Application	Disease	Source	Sample Type
Hepatol 1994, [ref] Trinchet JC. et al. [80]	Bile acids	Protein	Diagnosis biomarker	ASH	-	Serum
PLoS One 2015, Moratti, Vezzalini et al. [81]	sPTPRG	Protein	Prognosis biomarker	AH	-	Plasma
J Transl Med 2015, Momen-Heravi, Saha et al. [83]	miR-192 and miR-30a	Exosomes	Predictor biomarker	AH	-	Serum
JCI Insight 2017, Cai, Xu et al. [86]	mtDNA	Microparticles	Prognosis biomarker	AH	-	Serum
Hepatology 2018, Saha, Momen-Heravi et al. [87]	CCL2	EVs	Prognosis biomarker	AH	-	Mouse model Serum
PLoS One 2017, Cho, Im et al. [89]	Liver-specific proteins	EVs	Prognosis biomarker	AH	Hepatocytes	plasma
Aliment Pharmacol Ther 2018, Sukriti, Maras et al. [91]	CD34+ and ASGPR+	MVs	Prognosis biomarker	AH	haematopoietic stem-cells, hepatocytes.	Serum
Journal of Hepatology 2016, Verma VK. et al. [92]	CD40L	EVs	Prognosis biomarker	AH	Hepatocytes	Serum
Sci Rep 2017, Lamichhane, Leung et al. [98]	lncRNA-HOTAIR lncRNA-MALAT1	EVs	Prognosis biomarker	AH	Endothelial cells	Mouse model Serum
Hepatology 2015, Chang, Xu et al. [58]	CXCL1	-	Prognosis biomarker	ALD	-	Serum

Protein tyrosine phosphatase receptor gamma extracellular domain (sPTPRG); Alanine transaminase (ALT); alcoholic hepatitis (AH); alcoholic liver disease (ALD); alcoholic steatohepatitis (ASH); chemokine (C-C motif) ligand 2 (CCL2); chemokine (C-X-C motif) ligand 1 (CXCL1); extracellular vesicle (EV); Microvesicles (MVs); long non-coding RNA (lncRNA); microRNA (miR); mitochondrial DNA (mtDNA).

## Abbreviations

ALT	alanine transaminase
AH	alcoholic hepatitis
ALD	alcoholic liver disease
ASH	alcoholic steatohepatitis
cfDNA	cell-free DNA
CCL2	chemokine (C-C motif) ligand 2
CXCL1	chemokine (C-X-C motif) ligand 1
CHB	chronic hepatitis B virus
CHC	chronic hepatitis C virus
cccDNA	covalently closed circular DNA
dsDNA	double-stranded DNA
ESCRT	endosomal sorting complexes required for transport
EV	extracellular vesicle
GWAS	genome-wide association study
HSC	hepatic stellate cell
HAV	hepatitis A virus
HBV	hepatitis B virus
HCV	hepatitis C virus
HGV	hepatitis G virus
HCC	hepatocellular carcinoma
Hcy	homocysteine
HuH	human hepatocyte-derived carcinoma cell
ILV	intraluminal vesicle
lncRNA	long non-coding RNA
LPC	lysophosphatidylcholine
LPC	lysophosphatidylcholine
MSC	mesenchymal stem cell
mRNA	messenger RNA
MICA	MHC class I chain-related A
MPs/MVs	microparticles/microvesicles
miRNA	microRNA
mtDNA	mitochondrial DNA
MVB	multivesicular body
NAFLD	non-alcoholic fatty liver disease
NASH	non-alcoholic steatohepatitis
PA	palmitic acid
PBMC	peripheral blood mononuclear cell
PPARγ	peroxisome proliferator-activated receptor gamma
pgRNA	pre-genomic RNA
SNP	single nucleotide polymorphism
ssDNA	single-stranded DNA
sMICA	soluble MICA
S1P	sphingosine 1 phosphate
SK2	sphingosine kinase 2
TLR9	toll-like receptor 9
TAG	triacylglycerol
TG	triacylglycerol
TNFRSF10B	tumor necrosis factor receptor superfamily member 10B
VLDL	very-low-density lipoprotein
HSPG	heparan sulfate proteoglycans
L-SIGN	liver/lymph node-specific intracellular adhesion molecules-3 grabbing non-integrin
DC-SIGN	dendritic cell–specific ICAM-3 grabbing-nonintegrin
LDL-R	low-density lipoprotein receptor
TfR1	transferrin receptor
NPC1L1	NPC1-like intracellular cholesterol transporter 1
CD81	cluster of differentiation 81
CLDN-1	claudin 1
SR-B1	scavenger receptor class B type 1
OCLN	occludin
Ago2	argonaute RISC catalytic component 2
ACC	acetyl-CoA carboxylase
ALKP	alkaline phosphatase
TNFα	tumor necrosis factor alpha
Tsg101	tumor susceptibility 101

## References

[1] Asrani S.K., Devarbhavi H., Eaton J., Kamath P.S. (2019). Burden of liver diseases in the world. J. Hepatol..

[2] Perumpail B.J., Khan M.A., Yoo E.R., Cholankeril G., Kim D., Ahmed A. (2017). Clinical epidemiology and disease burden of nonalcoholic fatty liver disease. World J. Gastroenterol..

[3] Hsu P.Y., Hsu C.T., Yeh M.L., Huang C.F., Huang C.I., Liang P.C., Lin Y.H., Hsieh M.Y., Wei Y.J., Hsieh M.H. (2019). Early fibrosis but late tumor stage and worse outcomes in hepatocellular carcinoma patients without hepatitis B or hepatitis C. Dig. Dis. Sci..

[4] Jun T.W., Yeh M.L., Yang J.D., Chen V.L., Nguyen P., Giama N.H., Huang C.F., Hsing A.W., Dai C.Y., Huang J.F. (2018). More advanced disease and worse survival in cryptogenic compared to viral hepatocellular carcinoma. Liver Int..

[5] Lindenmeyer C.C., McCullough A.J. (2018). The natural history of nonalcoholic fatty liver disease-an evolving view. Clin. Liver. Dis..

[6] Horowitz J.M., Venkatesh S.K., Ehman R.L., Jhaveri K., Kamath P., Ohliger M.A., Samir A.E., Silva A.C., Taouli B., Torbenson M.S. (2017). Evaluation of hepatic fibrosis: A review from the society of abdominal radiology disease focus panel. Abdom. Radiol. (NY).

[7] Ratner M. (2018). Jury out on liquid biopsies for cancer. Nat. Biotechnol..

[8] Konoshenko M.Y., Lekchnov E.A., Vlassov A.V., Laktionov P.P. (2018). Isolation of extracellular vesicles: General methodologies and latest trends. Biomed. Res. Int..

[9] Cocucci E., Meldolesi J. (2015). Ectosomes and exosomes: Shedding the confusion between extracellular vesicles. Trends Cell. Biol..

[10] Kowal J., Arras G., Colombo M., Jouve M., Morath J.P., Primdal-Bengtson B., Dingli F., Loew D., Tkach M., Théry C. (2016). Proteomic comparison defines novel markers to characterize heterogeneous populations of extracellular vesicle subtypes. Proc. Natl. Acad. Sci. USA.

[11] Barile L., Vassalli G. (2017). Exosomes: Therapy delivery tools and biomarkers of diseases. Pharmacol. Ther..

[12] Conde-Vancells J., Rodriguez-Suarez E., Embade N., Gil D., Matthiesen R., Valle M., Elortza F., Lu S.C., Mato J.M., Falcon-Perez J.M. (2008). Characterization and comprehensive proteome profiling of exosomes secreted by hepatocytes. J. Proteom. Res..

[13] Sung S., Kim J., Jung Y. (2018). Liver-derived exosomes and their implications in liver pathobiology. Int. J. Mol. Sci..

[14] Momen-Heravi F., Bala S., Kodys K., Szabo G. (2015). Exosomes derived from alcohol-treated hepatocytes horizontally transfer liver specific miRNA-122 and sensitize monocytes to LPS. Sci. Rep..

[15] Izquierdo-Useros N., Puertas M.C., Borràs F.E., Blanco J., Martinez-Picado J. (2011). Exosomes and retroviruses: The chicken or the egg?. Cell Microbiol..

[16] Grove J., Marsh M. (2011). The cell biology of receptor-mediated virus entry. J. Cell Biol..

[17] Moller-Tank S., Kondratowicz A.S., Davey R.A., Rennert P.D., Maury W. (2013). Role of the phosphatidylserine receptor TIM-1 in enveloped-virus entry. J. Virol..

[18] Fitzner D., Schnaars M., van Rossum D., Krishnamoorthy G., Dibaj P., Bakhti M., Regen T., Hanisch U.K., Simons M. (2011). Selective transfer of exosomes from oligodendrocytes to microglia by macropinocytosis. J. Cell Sci..

[19] Feng D., Zhao W.L., Ye Y.Y., Bai X.C., Liu R.Q., Chang L.F., Zhou Q., Sui S.F. (2010). Cellular internalization of exosomes occurs through phagocytosis. Traffic (Cph. Den.).

[20] Miyanishi M., Tada K., Koike M., Uchiyama Y., Kitamura T., Nagata S. (2007). Identification of Tim4 as a phosphatidylserine receptor. Nature.

[21] Feng Z., Hensley L., McKnight K.L., Hu F., Madden V., Ping L., Jeong S.H., Walker C., Lanford R.E., Lemon S.M. (2013). A pathogenic picornavirus acquires an envelope by hijacking cellular membranes. Nature.

[22] McKnight K.L., Xie L., Gonzalez-Lopez O., Rivera-Serrano E.E., Chen X., Lemon S.M. (2017). Protein composition of the hepatitis A virus quasi-envelope. Proc. Natl. Acad. Sci. USA.

[23] Nagashima S., Jirintai S., Takahashi M., Kobayashi T., Tanggis, Nishizawa T., Kouki T., Yashiro T., Okamoto H. (2014). Hepatitis E virus egress depends on the exosomal pathway, with secretory exosomes derived from multivesicular bodies. J. Gen. Virol..

[24] Lindenbach B.D., Rice C.M. (2013). The ins and outs of hepatitis C virus entry and assembly. Nat. Rev. Microbiol..

[25] Bukong T.N., Momen-Heravi F., Kodys K., Bala S., Szabo G. (2014). Exosomes from hepatitis C infected patients transmit HCV infection and contain replication competent viral RNA in complex with Ago2-miR122-HSP90. PLoS Pathog..

[26] Meuleman P., Hesselgesser J., Paulson M., Vanwolleghem T., Desombere I., Reiser H., Leroux-Roels G. (2008). Anti-CD81 antibodies can prevent a hepatitis C virus infection in vivo. Hepatology.

[27] Harman A.N., Nasr N., Feetham A., Galoyan A., Alshehri A.A., Rambukwelle D., Botting R.A., Hiener B.M., Diefenbach E., Diefenbach R.J. (2015). HIV blocks interferon induction in human dendritic cells and macrophages by dysregulation of TBK1. J. Virol..

[28] Bhattarai N., McLinden J.H., Xiang J., Landay A.L., Chivero E.T., Stapleton J.T. (2013). GB virus C particles inhibit T cell activation via envelope E2 protein-mediated inhibition of TCR signaling. J. Immunol..

[29] Akinyemiju T., Abera S., Ahmed M., Alam N., Alemayohu M.A., Allen C., Al-Raddadi R., Alvis-Guzman N., Amoako Y., Global Burden of Disease Liver Cancer Collaboration (2017). The burden of primary liver cancer and underlying etiologies from 1990 to 2015 at the global, regional, and national level: Results from the Global Burden of Disease Study 2015. JAMA Oncol..

[30] Saha B., Kodys K., Szabo G. (2016). Hepatitis C virus-induced monocyte differentiation into polarized M2 macrophages promotes stellate cell activation via TGF-beta. Cell Mol. Gastroenterol. Hepatol..

[31] Fusegawa H., Shiraishi K., Ogasawara F., Shimizu M., Haruki Y., Miyachi H., Matsuzaki S., Ando Y. (2002). Platelet activation in patients with chronic hepatitis C. Tokai J. Exp. Clin. Med..

[32] Kornek M., Lynch M., Mehta S.H., Lai M., Exley M., Afdhal N.H., Schuppan D. (2012). Circulating microparticles as disease-specific biomarkers of severity of inflammation in patients with hepatitis C or nonalcoholic steatohepatitis. Gastroenterology.

[33] Li J., Liu K., Liu Y., Xu Y., Zhang F., Yang H., Liu J., Pan T., Chen J., Wu M. (2013). Exosomes mediate the cell-to-cell transmission of IFN-alpha-induced antiviral activity. Nat. Immunol..

[34] Ramakrishnaiah V., Thumann C., Fofana I., Habersetzer F., Pan Q., de Ruiter P.E., Willemsen R., Demmers J.A., Stalin Raj V., Jenster G. (2013). Exosome-mediated transmission of hepatitis C virus between human hepatoma Huh7.5 cells. Proc. Natl. Acad. Sci. USA.

[35] Szabo G., Momen-Heravi F. (2017). Extracellular vesicles in liver disease and potential as biomarkers and therapeutic targets. Nat. Rev. Gastroenterol. Hepatol..

[36] Xu H., Dong X., Chen Y., Wang X. (2018). Serum exosomal hnRNPH1 mRNA as a novel marker for hepatocellular carcinoma. Clin. Chem. Lab. Med..

[37] Ruan L., Huang L., Zhao L., Wang Q., Pan X., Zhang A., Bai Q., Lv Z. (2018). The Interaction of lncRNA-HEIH and lncRNA-HULC with HBXIP in hepatitis B patients. Gastroenterol. Res. Pract..

[38] Liu Y., Feng J., Sun M., Yang G., Yuan H., Wang Y., Bu Y., Zhao M., Zhang S., Zhang X. (2019). Long non-coding RNA HULC activates HBV by modulating HBx/STAT3/miR-539/APOBEC3B signaling in HBV-related hepatocellular carcinoma. Cancer Lett..

[39] Zhang C., Yang X., Qi Q., Gao Y., Wei Q., Han S. (2018). lncRNA-HEIH in serum and exosomes as a potential biomarker in the HCV-related hepatocellular carcinoma. Cancer Biomark..

[40] Lo P.H., Urabe Y., Kumar V., Tanikawa C., Koike K., Kato N., Miki D., Chayama K., Kubo M., Nakamura Y. (2013). Identification of a functional variant in the MICA promoter which regulates MICA expression and increases HCV-related hepatocellular carcinoma risk. PLoS ONE.

[41] Huang C.F., Wang S.C., Yeh M.L., Huang C.I., Tsai P.C., Lin Z.Y., Chen S.C., Dai C.Y., Huang J.F., Chuang W.L. (2019). Association of serial serum major histocompatibility complex class I chain-related A measurements with hepatocellular carcinoma in chronic hepatitis C patients after viral eradication. J. Gastroenterol. Hepatol..

[42] Huang C.F., Wang S.C., Chang W.T., Yeh M.L., Huang C.I., Lin Z.Y., Chen S.C., Chuang W.L., Huang J.F., Dai C.Y. (2018). Lower protein expression levels of MHC class I chain-related gene A in hepatocellular carcinoma are at high risk of recurrence after surgical resection. Sci. Rep..

[43] Kumar V., Yi Lo P.H., Sawai H., Kato N., Takahashi A., Deng Z., Urabe Y., Mbarek H., Tokunaga K., Tanaka Y. (2012). Soluble MICA and a MICA variation as possible prognostic biomarkers for HBV-induced hepatocellular carcinoma. PLoS ONE.

[44] Allweiss L., Dandri M. (2017). The role of cccDNA in HBV maintenance. Viruses.

[45] Lin N., Ye A., Lin J., Liu C., Huang J., Fu Y., Wu S., Xu S., Wang L., Ou Q. (2020). Diagnostic value of serum pgRNA detection in HBV-infected patients with different clinical outcomes. J. Clin. Microbiol..

[46] Tokuhisa Y., Iizuka N., Sakaida I., Moribe T., Fujita N., Miura T., Tamatsukuri S., Ishitsuka H., Uchida K., Terai S. (2007). Circulating cell-free DNA as a predictive marker for distant metastasis of hepatitis C virus-related hepatocellular carcinoma. Br. J. Cancer.

[47] Iida M., Iizuka N., Sakaida I., Moribe T., Fujita N., Miura T., Tamatsukuri S., Ishitsuka H., Uchida K., Terai S. (2008). Relation between serum levels of cell-free DNA and inflammation status in hepatitis C virus-related hepatocellular carcinoma. Oncol. Rep..

[48] Ng C.K.Y., Di Costanzo G.G., Terracciano L.M., Piscuoglio S. (2018). Circulating cell-free DNA in hepatocellular carcinoma: Current insights and outlook. Front. Med. (Lausanne).

[49] Brumbaugh D.E., Friedman J.E. (2014). Developmental origins of nonalcoholic fatty liver disease. Pediatr. Res..

[50] Pierantonelli I., Svegliati-Baroni G. (2019). Nonalcoholic fatty liver disease: Basic pathogenetic mechanisms in the progression from NAFLD to NASH. Transplantation.

[51] Chan S.L., Wong A.M., Lee K., Wong N., Chan A.K. (2016). Personalized therapy for hepatocellular carcinoma: Where are we now?. Cancer Treat. Rev..

[52] Yang R.X., Hu C.X., Sun W.L., Pan Q., Shen F., Yang Z., Su Q., Xu G.W., Fan J.G. (2017). Serum monounsaturated triacylglycerol predicts steatohepatitis in patients with non-alcoholic fatty liver disease and chronic hepatitis B. Sci. Rep..

[53] Fartoux L., Chazouillères O., Wendum D., Poupon R., Serfaty L. (2005). Impact of steatosis on progression of fibrosis in patients with mild hepatitis C. Hepatology.

[54] Mehta S.R., Thomas E.L., Bell J.D., Johnston D.G., Taylor-Robinson S.D. (2008). Non-invasive means of measuring hepatic fat content. World J. Gastroenterol..

[55] Hardy T., Zeybel M., Day C.P., Dipper C., Masson S., McPherson S., Henderson E., Tiniakos D., White S., French J. (2017). Plasma DNA methylation: A potential biomarker for stratification of liver fibrosis in non-alcoholic fatty liver disease. Gut.

[56] De Carvalho S.C., Muniz M.T., Siqueira M.D., Siqueira E.R., Gomes A.V., Silva K.A., Bezerra L.C., D’Almeida V., de Oliveira C.P., Pereira L.M. (2013). Plasmatic higher levels of homocysteine in non-alcoholic fatty liver disease (NAFLD). Nutr. J..

[57] Gulsen M., Yesilova Z., Bagci S., Uygun A., Ozcan A., Ercin C.N., Erdil A., Sanisoglu S.Y., Cakir E., Ates Y. (2005). Elevated plasma homocysteine concentrations as a predictor of steatohepatitis in patients with non-alcoholic fatty liver disease. J. Gastroenterol. Hepatol..

[58] Chang B., Xu M.J., Zhou Z., Cai Y., Li M., Wang W., Feng D., Bertola A., Wang H., Kunos G. (2015). Short- or long-term high-fat diet feeding plus acute ethanol binge synergistically induce acute liver injury in mice: An important role for CXCL1. Hepatology.

[59] Chashmniam S., Ghafourpour M., Rezaei Farimani A., Gholami A., Nobakht Motlagh Ghoochani B.F. (2019). Metabolomic Biomarkers in the Diagnosis of Non-Alcoholic Fatty Liver Disease. Hepat. Mon..

[60] Morgan K., Uyuni A., Nandgiri G., Mao L., Castaneda L., Kathirvel E., French S.W., Morgan T.R. (2008). Altered expression of transcription factors and genes regulating lipogenesis in liver and adipose tissue of mice with high fat diet-induced obesity and nonalcoholic fatty liver disease. Eur. J. Gastroenterol. Hepatol..

[61] Yoshida M. (2011). Novel role of NPC1L1 in the regulation of hepatic metabolism: Potential contribution of ezetimibe in NAFLD/NASH treatment. Curr. Vasc. Pharmacol..

[62] Zhao Y., Zhao M.F., Jiang S., Wu J., Liu J., Yuan X.W., Shen D., Zhang J.Z., Zhou N., He J. (2020). Liver governs adipose remodeling via extracellular vesicles in response to lipid overload. Nat. Commun..

[63] Ibrahim S.H., Hirsova P., Tomita K., Bronk S.F., Werneburg N.W., Harrison S.A., Goodfellow V.S., Malhi H., Gores G.J. (2016). Mixed lineage kinase 3 mediates release of C-X-C motif ligand 10-bearing chemotactic extracellular vesicles from lipotoxic hepatocytes. Hepatology.

[64] Povero D., Eguchi A., Li H., Johnson C.D., Papouchado B.G., Wree A., Messer K., Feldstein A.E. (2014). Circulating extracellular vesicles with specific proteome and liver microRNAs are potential biomarkers for liver injury in experimental fatty liver disease. PLoS ONE.

[65] Hirsova P., Ibrahim S.H., Krishnan A., Verma V.K., Bronk S.F., Werneburg N.W., Charlton M.R., Shah V.H., Malhi H., Gores G.J. (2016). Lipid-induced signaling causes release of inflammatory extracellular vesicles from hepatocytes. Gastroenterology.

[66] Garcia-Martinez I., Santoro N., Chen Y., Hoque R., Ouyang X., Caprio S., Shlomchik M.J., Coffman R.L., Candia A., Mehal W.Z. (2016). Hepatocyte mitochondrial DNA drives nonalcoholic steatohepatitis by activation of TLR9. J. Clin. Investig..

[67] Tosello-Trampont A.C., Landes S.G., Nguyen V., Novobrantseva T.I., Hahn Y.S. (2012). Kuppfer cells trigger nonalcoholic steatohepatitis development in diet-induced mouse model through tumor necrosis factor-alpha production. J. Biol. Chem..

[68] Ibrahim S.H., Gores G.J., Hirsova P., Kirby M., Miles L., Jaeschke A., Kohli R. (2014). Mixed lineage kinase 3 deficient mice are protected against the high fat high carbohydrate diet-induced steatohepatitis. Liver Int..

[69] Mridha A.R., Haczeyni F., Yeh M.M., Haigh W.G., Ioannou G.N., Barn V., Ajamieh H., Adams L., Hamdorf J.M., Teoh N.C. (2017). TLR9 is up-regulated in human and murine NASH: Pivotal role in inflammatory recruitment and cell survival. Clin. Sci. (Lond.).

[70] Lee Y.S., Kim S.Y., Ko E., Lee J.H., Yi H.S., Yoo Y.J., Je J., Suh S.J., Jung Y.K., Kim J.H. (2017). Exosomes derived from palmitic acid-treated hepatocytes induce fibrotic activation of hepatic stellate cells. Sci. Rep..

[71] Witek R.P., Yang L., Liu R., Jung Y., Omenetti A., Syn W.K., Choi S.S., Cheong Y., Fearing C.M., Agboola K.M. (2009). Liver cell-derived microparticles activate hedgehog signaling and alter gene expression in hepatic endothelial cells. Gastroenterology.

[72] Deng Z.B., Liu Y., Liu C., Xiang X., Wang J., Cheng Z., Shah S.V., Zhang S., Zhang L., Zhuang X. (2009). Immature myeloid cells induced by a high-fat diet contribute to liver inflammation. Hepatology.

[73] Harrell C.R., Jovicic N., Djonov V., Arsenijevic N., Volarevic V. (2019). Mesenchymal stem cell-derived exosomes and other extracellular vesicles as new remedies in the therapy of inflammatory diseases. Cells.

[74] Eguchi A., Mulya A., Lazic M., Radhakrishnan D., Berk M.P., Povero D., Gornicka A., Feldstein A.E. (2015). Microparticles release by adipocytes act as “find-me” signals to promote macrophage migration. PLoS ONE.

[75] Kakazu E., Mauer A.S., Yin M., Malhi H. (2016). Hepatocytes release ceramide-enriched pro-inflammatory extracellular vesicles in an IRE1alpha-dependent manner. J. Lipid. Res..

[76] Gaggini M., Carli F., Rosso C., Younes R., D’Aurizio R., Bugianesi E., Gastaldelli A. (2019). Altered Metabolic Profile and Adipocyte Insulin Resistance Mark Severe Liver Fibrosis in Patients with Chronic Liver Disease. Int. J. Mol. Sci..

[77] Gao B., Bataller R. (2011). Alcoholic liver disease: Pathogenesis and new therapeutic targets. Gastroenterology.

[78] Gao B., Tsukamoto H. (2016). Inflammation in alcoholic and nonalcoholic fatty liver disease: Friend or foe?. Gastroenterology.

[79] Mills S.J., Harrison S.A. (2005). Comparison of the natural history of alcoholic and nonalcoholic fatty liver disease. Curr. Gastroenterol. Rep..

[80] Trinchet J.C., Gerhardt M.F., Balkau B., Munz C., Poupon R.E. (1994). Serum bile acids and cholestasis in alcoholic hepatitis. Relationship with usual liver tests and histological features. J. Hepatol..

[81] Moratti E., Vezzalini M., Tomasello L., Giavarina D., Sorio C. (2015). Identification of protein tyrosine phosphatase receptor gamma extracellular domain (sPTPRG) as a natural soluble protein in plasma. PLoS ONE.

[82] Xu M.J., Cai Y., Wang H., Altamirano J., Chang B., Bertola A., Odena G., Lu J., Tanaka N., Matsusue K. (2015). Fat-specific protein 27/CIDEC promotes development of alcoholic steatohepatitis in mice and humans. Gastroenterology.

[83] Momen-Heravi F., Saha B., Kodys K., Catalano D., Satishchandran A., Szabo G. (2015). Increased number of circulating exosomes and their microRNA cargos are potential novel biomarkers in alcoholic hepatitis. J. Tranl. Med..

[84] Cho Y.E., Mezey E., Hardwick J.P., Salem N., Clemens D.L., Song B.J. (2017). Increased ethanol-inducible cytochrome P450-2E1 and cytochrome P450 isoforms in exosomes of alcohol-exposed rodents and patients with alcoholism through oxidative and endoplasmic reticulum stress. Hepatol. Commun..

[85] Bode C., Kugler V., Bode J.C. (1987). Endotoxemia in patients with alcoholic and non-alcoholic cirrhosis and in subjects with no evidence of chronic liver disease following acute alcohol excess. J. Hepatol..

[86] Cai Y., Xu M.J., Koritzinsky E.H., Zhou Z., Wang W., Cao H., Yuen P.S., Ross R.A., Star R.A., Liangpunsakul S. (2017). Mitochondrial DNA-enriched microparticles promote acute-on-chronic alcoholic neutrophilia and hepatotoxicity. JCI Insight.

[87] Saha B., Momen-Heravi F., Furi I., Kodys K., Catalano D., Gangopadhyay A., Haraszti R., Satishchandran A., Iracheta-Vellve A., Adejumo A. (2018). Extracellular vesicles from mice with alcoholic liver disease carry a distinct protein cargo and induce macrophage activation through heat shock protein 90. Hepatology.

[88] Rodríguez-Suárez E., Gonzalez E., Hughes C., Conde-Vancells J., Rudella A., Royo F., Palomo L., Elortza F., Lu S.C., Mato J.M. (2014). Quantitative proteomic analysis of hepatocyte-secreted extracellular vesicles reveals candidate markers for liver toxicity. J. Proteom..

[89] Cho Y.E., Im E.J., Moon P.G., Mezey E., Song B.J., Baek M.C. (2017). Increased liver-specific proteins in circulating extracellular vesicles as potential biomarkers for drug- and alcohol-induced liver injury. PLoS ONE.

[90] Bissonnette J., Altamirano J., Devue C., Roux O., Payancé A., Lebrec D., Bedossa P., Valla D., Durand F., Ait-Oufella H. (2017). A prospective study of the utility of plasma biomarkers to diagnose alcoholic hepatitis. Hepatology.

[91] Sukriti S., Maras J.S., Bihari C., Das S., Vyas A.K., Sharma S., Hussain S., Shasthry S., Choudhary A., Premkumar M. (2018). Microvesicles in hepatic and peripheral vein can predict nonresponse to corticosteroid therapy in severe alcoholic hepatitis. Aliment Pharmacol. Ther..

[92] Verma V.K., Li H., Wang R., Hirsova P., Mushref M., Liu Y., Cao S., Contreras P.C., Malhi H., Kamath P.S. (2016). Alcohol stimulates macrophage activation through caspase-dependent hepatocyte derived release of CD40L containing extracellular vesicles. J. Hepatol..

[93] Kobayashi Y. (2008). The role of chemokines in neutrophil biology. Front. Biosci..

[94] Bautista A.P. (2002). Neutrophilic infiltration in alcoholic hepatitis. Alcohol.

[95] Dominguez M., Miquel R., Colmenero J., Moreno M., García-Pagán J.C., Bosch J., Arroyo V., Ginès P., Caballería J., Bataller R. (2009). Hepatic expression of CXC chemokines predicts portal hypertension and survival in patients with alcoholic hepatitis. Gastroenterology.

[96] Sheron N., Bird G., Koskinas J., Portmann B., Ceska M., Lindley I., Williams R. (1993). Circulating and tissue levels of the neutrophil chemotaxin interleukin-8 are elevated in severe acute alcoholic hepatitis, and tissue levels correlate with neutrophil infiltration. Hepatology.

[97] Roh Y.S., Zhang B., Loomba R., Seki E. (2015). TLR2 and TLR9 contribute to alcohol-mediated liver injury through induction of CXCL1 and neutrophil infiltration. Am. J. Physiol. Gastrointest. Liver Physiol..

[98] Lamichhane T.N., Leung C.A., Douti L.Y., Jay S.M. (2017). Ethanol induces enhanced vascularization bioactivity of endothelial cell-derived extracellular vesicles via regulation of microRNAs and long non-coding RNAs. Sci. Rep..

